# Influence of cell type specific infectivity and tissue composition on SARS-CoV-2 infection dynamics within human airway epithelium

**DOI:** 10.1371/journal.pcbi.1011356

**Published:** 2023-08-11

**Authors:** Benjamin Raach, Nils Bundgaard, Marika J. Haase, Jörn Starruß, Rocio Sotillo, Megan L. Stanifer, Frederik Graw

**Affiliations:** 1 BioQuant-Center for Quantitative Biology, Heidelberg University, Heidelberg, Germany; 2 Center for Information Services and High Performance Computing, TU Dresden, Dresden, Germany; 3 Division of Molecular Thoracic Oncology, German Cancer Research Center (DKFZ), Heidelberg, Germany; 4 Department of Infectious Diseases, Molecular Virology, University Hospital Heidelberg, Heidelberg, Germany; 5 University of Florida, College of Medicine, Dept. of Molecular Genetics and Microbiology, Gainesville, Florida, United States of America; 6 Interdisciplinary Center for Scientific Computing, Heidelberg University, Heidelberg, Germany; 7 Friedrich-Alexander-Universität Erlangen-Nürnberg, Department of Medicine 5, Erlangen, Germany; University of Pittsburgh, UNITED STATES

## Abstract

Human airway epithelium (HAE) represents the primary site of viral infection for SARS-CoV-2. Comprising different cell populations, a lot of research has been aimed at deciphering the major cell types and infection dynamics that determine disease progression and severity. However, the cell type-specific replication kinetics, as well as the contribution of cellular composition of the respiratory epithelium to infection and pathology are still not fully understood. Although experimental advances, including Air-liquid interface (ALI) cultures of reconstituted pseudostratified HAE, as well as lung organoid systems, allow the observation of infection dynamics under physiological conditions in unprecedented level of detail, disentangling and quantifying the contribution of individual processes and cells to these dynamics remains challenging. Here, we present how a combination of experimental data and mathematical modelling can be used to infer and address the influence of cell type specific infectivity and tissue composition on SARS-CoV-2 infection dynamics. Using a stepwise approach that integrates various experimental data on HAE culture systems with regard to tissue differentiation and infection dynamics, we develop an individual cell-based model that enables investigation of infection and regeneration dynamics within pseudostratified HAE. In addition, we present a novel method to quantify tissue integrity based on image data related to the standard measures of transepithelial electrical resistance measurements. Our analysis provides a first aim of quantitatively assessing cell type specific infection kinetics and shows how tissue composition and changes in regeneration capacity, as e.g. in smokers, can influence disease progression and pathology. Furthermore, we identified key measurements that still need to be assessed in order to improve inference of cell type specific infection kinetics and disease progression. Our approach provides a method that, in combination with additional experimental data, can be used to disentangle the complex dynamics of viral infection and immunity within human airway epithelial culture systems.

## Author summary

The cells of the human airway epithelium are one of the first cells that get into contact with SARS-CoV-2 during infection. To which extent the different cell types that reconstitute the epithelium contribute to the infection dynamics and also influence tissue pathology and regeneration is still not fully understood. While advanced experimental systems, such as air-liquid interface (ALI) cultures of reconstituted human airway epithelium, allow a detailed experimental investigation of the infection dynamics under physiologically relevant conditions, disentangling the contribution of the various intra- and intercellular processes to the progression of infection and disease severity remains complicated. Here, we combined experimental data on SARS-CoV-2 infection within ALI-cultures with a detailed individual-cell based model of the pseudostratified epithelium to reveal and quantify the role of cell-type specific infection kinetics for disease progression and tissue integrity. We can show how tissue composition and altered cell differentiation dynamics affect the regeneration capacity of the airway epithelium and, thus, disease severity. We highlight key processes that still need to be determined in order to reliably assess the interplay between cell differentiation, infection and pathology during SARS-CoV-2 infection, and provide a method to quantitatively evaluate infection dynamics within tissues.

## Introduction

Deciphering the main target cells and viral replication dynamics of the novel SARS-CoV-2 corona virus is an essential prerequisite for understanding infection kinetics and disease progression within patients. Being mainly transmitted via the respiratory route, the human airway epithelium (HAE) represents the primary site of viral infection for SARS-CoV-2 [1,2]. Once inhaled and entering the respiratory system, viral particles infect cells of the airway epithelium and release new progeny viruses propagating this infection throughout the tissue.

HAE is composed of different cell types that form a pseudostratified epithelium with each of them contributing to the functionality of the tissue [3,4]: Basal cells that are located close to the basal membrane represent a stem-cell like population of the tissue, which proliferate and differentiate into other cell types, enabling tissue regeneration upon injury [3,5]. Secretory cells, such as club and goblet cells, secrete mucous that contributes to epithelial protection by engulfing inhaled particles and microbes. Removal of these particles is then mediated by ciliated cells, the third major cell type of the HAE. These cells contain numerous cilia on their surface which beat rhythmically to allow a coordinated transport of the mucous out of the respiratory tract [6].

Human airway epithelial cells line all parts of the respiratory system including the nasal cavity and throat of the upper respiratory tract, as well as the trachea, bronchi and bronchioles of the lower respiratory tract. Each of these parts differs in the specific composition of the different cell types within the respective epithelium. Ciliated cells are the dominant cell population comprising between 50 to 70% of the cells [7], while secretory and basal cells comprise up to 11–36% and between 6–31% of the cells [3,8–10], respectively. Thereby, the actual frequency depends on the localization within the respiratory tree [7].

Since the beginning of the SARS-CoV-2 pandemic, a lot of effort has been made in deciphering the cellular and molecular details that determine infection kinetics and disease progression within tissues. Air-liquid-interface (ALI) cultures of human airway epithelium have been an essential tool for studying SARS-CoV-2 infection dynamics under physiological-like conditions [1,11–15]. By providing a pseudo-stratified epithelium grown from HAE cells, these cultures allow examining infection dynamics at various level of detail within manipulative conditions. However, disentangling the contribution of cell types and tissue composition to infection, as well as quantifying cell type specific infection kinetics is often difficult given the complexity of the various molecular and cellular processes involved and the available experimental measurements.

In this study, we present a method to combine experimental data of SARS-CoV-2 infection within HAE culture systems with mathematical modelling to quantify cell-type specific infectivity and to determine the interplay between disease pathology and tissue regeneration dynamics. To this end, we developed an individual cell-based model that provides a detailed representation of pseudo-stratified HAE-culture systems accounting for individual cell-types and their interactions. The model is based on the cellular Potts modelling framework [16] and explicitly accounts for a layered tissue structure, as well as target cell heterogeneity and dynamics in comparison to previous approaches modelling the spread of viral infection, and even SARS-CoV-2, within tissues [17–19]. By parameterizing the individual processes of cell differentiation, infection kinetics and cellular turnover using different published experimental measurements, as well as developing a method to quantify tissue integrity based on image data, we could investigate the role of individual cell types on infection dynamics and tissue pathology. With this we are able to show how differences in epithelial composition and regeneration capacity, as observed in nasal or smoking exposed tissue, influence infection and tissue pathology.

## Results

### Modelling cell turnover within Air-liquid interface cultures of human airway epithelium

**Fig 1:**
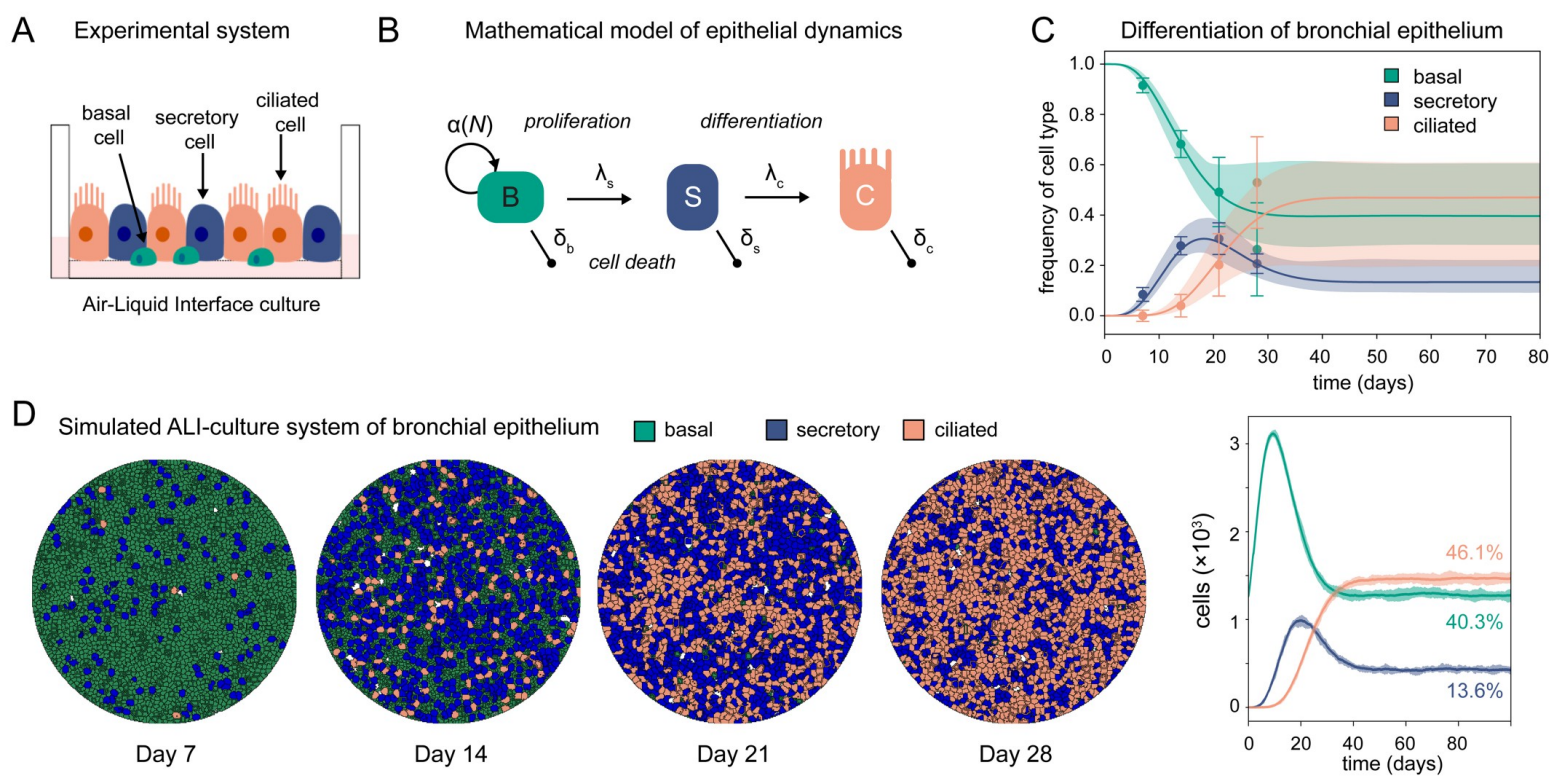
Cell differentiation dynamics in ALI-culture systems of human airway epithelium. (**A**) Sketch of human airway epithelial culture system. (**B**) Schematic of a mathematical model describing cell differentiation and turnover for human airway epithelial cell cultures distinguishing between basal (*B*), secretory (*S*) and ciliated (*C*) cells. For details see *Materials and Methods*. (**C**) Relative proportion of each cell type during differentiation of ALI-cultures of bronchial epithelium. Mean and standard deviation of the experimental data from [21] (dots/whiskers), with the predicted dynamics obtained when fitting the model shown in (**B**) to these data. The best fit (solid line) and 90%-prediction intervals for each cell type are shown (basal-green, secretory-blue, ciliated-orange). (**D**) Cellular Potts model of ALI-culture system mimicking pseudo-stratified epithelium at different time points during differentiation. Basal cells (green) are “overgrown” by secretory (blue) and ciliated (orange) cells. For graphical clarity, a reduced cell culture system is simulated starting with only ~1265 cells in total with *N*_max_ = 4085 cells. The average and range of *n* = 10 individual simulations using the best parameterization (**Table 1**) are shown with numbers indicating the average frequency of each cell type around day 80 post seeding (basal: 40.3% [39.3%, 42.5%], secretory: 13.6% [12.7%, 14.8%], ciliated: 46.1% [44.3%, 47.8%] (mean [min-max] across 10 simulations) (see also Movie1 at https://github.com/GrawLab/SARS-ALIculture).

To determine and evaluate how cell-type specific infectivity might influence SARS-CoV-2 infection within HAE cultures, we developed a mathematical model that describes the cellular turnover and tissue differentiation within the HAE-culture system (**Fig 1A**). The model distinguishes between the three main cell types observed within human airway epithelium, including basal (*B*), secretory (*S*) and ciliated (*C*) cells. Basal cells continuously proliferate at a rate *α* dependent on the total cell density in the system (*N = B+S+C*). They follow a linear differentiation pathway into secretory and subsequently ciliated cells at rate *λ*_*s*_ and *λ*_*c*_, respectively [20], with each cell type experiencing a cell type-specific death rate *δ*_*x*_ (**Fig 1B**, *Materials and Methods*). By fitting our model to experimental data on cell differentiation of ALI cultures of bronchial epithelial tissue [21], we estimated the rates of cellular turnover and differentiation, assuming that the system reaches confluence at around ~45 days after seeding (**Fig 1C** and **Table 1**). Given the complexity of the model and the available data, multiple parameter combinations are able to explain the observed dynamics with dependencies preventing individual parameter identifiability (**S1 Fig**). Nevertheless, we estimate consistently that basal cells have a longer half-life than ciliated and secretory cells, with the latter one having a ~3 (ciliated) to ~6 (basal)-fold shorter lifetime compared to the other two cell types (*t*_1/2*b*_ = 49.9 days vs. *t*_1/2*s*_ = 8.4 days vs. *t*_1/2*c*_ = 24.2 days). The observation that secretory cells have the shortest half-life of the considered cell types is in line with observations *in vivo* [20]. With our best parameterization, the bronchial epithelial tissue at confluence is dominated by ciliated cells, as generally observed [21], comprising ~46.9% [19.9%, 60.6%] of the cells, followed by basal (39.7% [28.4%, 60.2%]) and secretory cells (13.4% [9.4%, 22.0%]) (Numbers in squared brackets denote the 90%-prediction intervals).

**Table 1:** Parameter estimated for development and differentiation of bronchial epithelial ALI-culture systems. Parameters were estimated by fitting the model for epithelial differentiation dynamics (Eqs (1) and (2), **Fig 1B**) to the data for untreated ALI-cultures [21]. Values indicate best estimate and 90%-credible intervals (CrI) of estimates using an Approximate Bayesian Computation approach (pyABC, [23]) (see also **S1 Fig**).

Parameter	Unit	Value (90%-CrI)
Maximal proliferation rate of basal cells, *α*	d^-1^	0.34 (0.15, 0.77)
Differentiation rate basal-secretory, *λ*_*s*_	×10^−2^ d^-1^	8.41 (7.18, 9.86)
Differentiation rate secretory-ciliated, *λ*_*c*_	×10^−2^ d^-1^	16.23 (11.93, 21.10)
Loss rate of basal cells, *δ*_*b*_	×10^−2^ d^-1^	1.39 (0.10, 2.10)
Loss rate of secretory cells, *δ*_*s*_	×10^−2^ d^-1^	8.28 (3.01, 16.07)
Loss rate of ciliated cells, *δ*_*c*_	×10^−2^ d^-1^	2.86 (2.02, 6.99)
Maximal capacity of ALI-culture, *N*_*max*_	relative to *N*_*0*_ *= 1*	1.29 (1.08, 1.97)

Using the parameterization obtained above, we developed an individual cell-based model to account for the spatio-temporal dynamics of epithelial regeneration and cell differentiation. The model is based on a cellular Potts modeling (CPM) framework [22] which enables us to simulate inter- and intracellular dynamics on a local level by accounting for the polarized orientation of cells, i.e., basal and apical side of the epithelium, and varying morphologies of individual cells with increasing cell densities (**Fig 1D**). Our model is able to mimic the development of pseudo-stratified epithelium with basal cells slowly being overgrown by secretory and ciliated cells that will dominate the apical side of the culture (**Fig 1D,** see also Movie1 at https://github.com/GrawLab/SARS-ALIculture). Thus, we developed a model system that allows us to simulate and analyze the spatio-temporal dynamics of HAE cultures on a single-cell level.

### Determining cell-type specific SARS-CoV-2 infection dynamics within bronchial human airway epithelium

**Fig 2:**
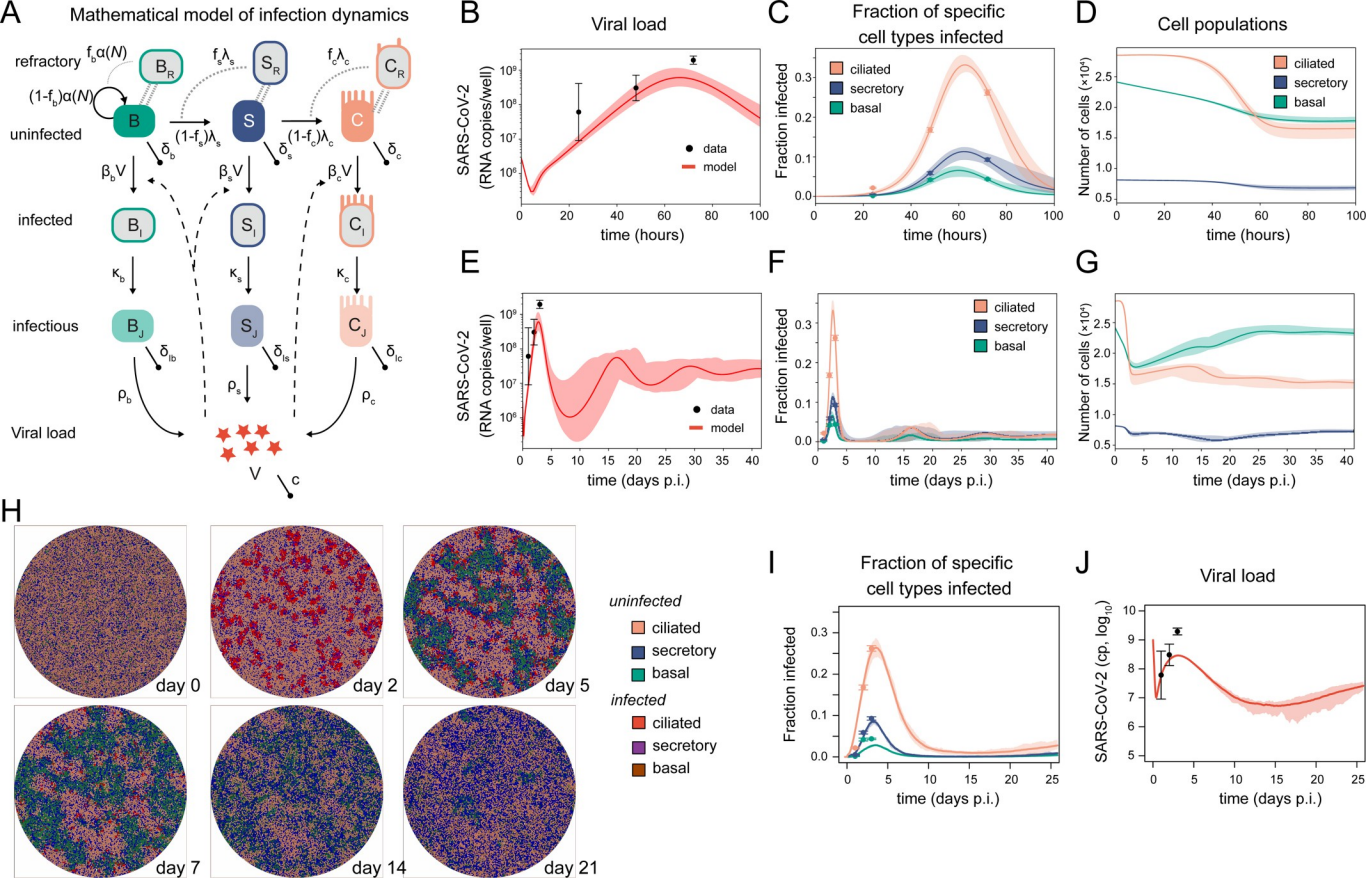
Cell-type specific infection kinetics. (**A**) Schematic of a viral dynamics model for SARS-CoV-2 infection within human airway epithelium considering uninfected (*X*), infected (*X*_*I*_) and infectious/productively infected cells (*X*_*J*_) for each of the different cell types *X*∈{*B*,*S*,*C*}. In addition, a population of cells being refractory to infection (*X*_*R*_) is considered for each cell type to account for spatial separation and possible innate immune protection of cells (grey arrows). (**B-D**) Measured (dots) and predicted (lines/shaded area) for the viral load (**B**), relative proportion of infected cell types (**C**) and total cell count for each cell type (**D**) by fitting the model shown in (**A**) to data on SARS-CoV-2 infection of ALI-cultures of bronchial epithelium analyzed by scRNA-seq [27] are shown. Model predictions for the best estimate (solid line) and prediction bands (shaded area) based on the 90%-credible intervals of parameter estimates in the last ABC-generation (**Table 3**) are shown. (**E-G**) Predicted long-term oscillatory dynamics of the individual components. (**H**) Snapshots of the individual-cell based model simulating SARS-CoV-2 infection dynamics within bronchial epithelial tissue considering ~4.7×10^4^ cells in total (see also Movie2 at https://github.com/GrawLab/SARS-ALIculture). (**I-J**) Relative proportion of infected cells (**I**) and viral load dynamics (**J**) with the mean (solid line) and maximal range (shaded area) over 10 independent simulated cultures.

To extend our framework for determining cell-type specific infection kinetics of SARS-CoV-2 among human airway epithelial cells and analyze their influence on the progression of infection, we followed a two-step approach. In a first step, we extended our cell population-based mathematical model (**Fig 1B**, **Eq (1)**) to additionally account for the infection dynamics (**Fig 2A**). Analogous to standard models of viral dynamics [24,25], we distinguish between uninfected (*X*), infected (*X*_*I*_) and infectious cells (*X*_*J*_). Each of the different cell types considered (basal, *B*, secretory, *S*, ciliated, *C*), is assumed to experience cell-type specific rates of cell infection *β*_*x*_, of turning infectious and productively infected *κ*_*x*_, viral production *ρ*_*x*_, and cell death of productively infected cells *δ*_*Ix*_, with *x*∈{*B*,*S*,*C*}. Infection occurs proportionally to the total viral load, *V*, which is cleared from the system at rate *c*. The model and the mathematical equations are explained in detail in *Materials and Methods*. As an extension of this standard model, and in accordance with previous observations [12,26], we considered a proportion of each of the considered cell types to become refractory to infection (**Fig 2A**). This accounts for the spatial separation of cells within the culture and within different cell layers (basal vs. secretory/ciliated) in the light of the well-mixed assumption of the model, as well as for cells that are protected from infection, e.g. by innate immune mechanisms [26] or low cilia expression [12].

To provide a first assessment of cell-type specific infection kinetics, the model was fitted to data published by Ravindra et al. [27] showing time-resolved measurements on viral load and single-cell sequencing analyses for ALI-culture systems of bronchial epithelial tissue infected with SARS-CoV-2 (**Fig 2B–2D**). In accordance with other studies [12,15], Ravindra et al. [27] found that ciliated cells represent the majority of infected cells, with ~26% of these cells being infected at 3 days post infection. While our standard model was not able to explain the experimental data as it would predict faster growth kinetics in the frequency of infected cells than observed experimentally when aiming to match viral load dynamics (*not shown*), the extended model with consideration of refractory cell populations provided an appropriate representation of the observed dynamics of the viral load (**Fig 2B**) and the relative proportions of cells being infected (**Fig 2C**). In accordance with the observation of ciliated cells being the main target of SARS-CoV2 infection [1,12,27], our model predicts a substantial, rapid loss of this cell type with only ~50% of them remaining at ~60 hours post infection (**Fig 2D**). Given tissue regeneration, the model would predict long-term oscillatory infection dynamics as they have been observed elsewhere [12,15] that would eventually saturate (**Fig 2E–2G**). Parameter estimates reveal an average duration of the viral eclipse phase of ~5h (2.9h, 20.0h) and an average lifetime of productively infected cells of ~12.9 h (6h, 28h) with no indicative differences between the individual cell types (**Table 2**). The largest differences between cell types are observed in the fraction of refractory cells, *f*, which is due to the model formulation. With the fraction of refractory cells, *f*, not being independently interpretable from the viral transmission rate, *β*, but the latter ones largely comparable between cell types (**Table 2**), infectivity is substantially reduced for basal cells compared to ciliated cells. While this could be due to the different spatial location of these cell types within the pseudo-stratified epithelium, the high fraction of refractory secretory cells compared to ciliated cells points towards a cell intrinsic difference in viral infectivity, with ciliated cells being more prone to infection.

**Table 2:** Parameter estimates for cell-type specific infectivity. Measurements on viral load and cell-type specific infection based on single-cell RNA sequencing data of SARS-CoV-2 infected ALI-cultures of human bronchial airway epithelium [27] were analyzed using the model shown in Eqs (3) and (4) and **Fig 2A**. The best parameter combination and the 90%-credible intervals (CrI) in the last generation of an Approximation Bayesian Computation approach (pyABC) after 50 generations are shown (*values from literature). Epithelial turnover dynamics were parameterized according to the best estimate shown in **Table 1** (see also **S2 Fig)**.

Parameter	Unit	Cell Type		
		Basal	Secretory	Ciliated
Viral transmission rate, *β*	-log_10_ h^-1^	9.36 (9.08, 9.63)	9.40 (9.21, 9.73)	9.43 (9.18, 9.70)
Rate of infected cells to turn productively infected, *κ*	h^-1^	0.19 (0.09, 0.30)	0.18 (0.05, 0.34)	0.22 (0.16, 0.31)
Viral production rate, *ρ*	log_10_ copies h^-1^	4.38 (4.05, 4.98)	4.61 (4.16, 5.00)	4.69 (4.32, 4.94)
Death rate of productively infected cells, *δ*_*I*_	×10^−2^ h^-1^	8.09 (4.08, 10.96)	7.70 (3.54, 16.50)	7.43 (5.75, 9.96)
Proportion of refractory cells, *f*		0.91 (0.90, 0.95)	0.86 (0.80, 0.90)	0.57 (0.51, 0.60)
**Additional infection parameters**				
Viral clearance rate, *c*	h^-1^	0.63* [29,30]		

To disentangle the contribution of spatial and cell-intrinsic effects on the infection dynamics, we incorporated the viral processes into our spatially-resolved model of the HAE culture system. Starting from the parameterization obtained by the ODE-approach (**Table 2**), we additionally adapted the transmission dynamics, as here no specific refractory populations are considered with the CPM explicitly accounting for the spatial separation of cells (see *Materials and Methods* for details on the adaptation process). Furthermore, in contrast to the ODE-approach which solely attributed infection spread to cell-free virus, the CPM particularly accounted for the spread of infection by direct cell-to-cell transmission within the model simulations, which has been found to be responsible for roughly 90% of the infections within tissues [28] (see also **S2 Table**). We used a step-wise approach considering different model assumptions to test their appropriateness in simultaneously explaining different observations on SARS-CoV-2 infection dynamics within tissues that comprise longitudinal measurements on specific cell-type frequencies and viral load dynamics [12,27] (see *Materials & Methods* for a detailed explanation). As a first result, we found that the spatial separation of cells was not sufficient to explain the different infection dynamics of ciliated and secretory, as well as basal cells. In addition, the spatially explicit model indicated the necessity of a substantially higher infectivity of ciliated in comparison to secretory and basal cells to match experimental observations. Furthermore, to explain the early rapid increase of the proportion of infected cells [27] in combination with the not fully destroyed tissue integrity at later time points [12], inclusion of a rate limiting process was needed to improve explaining the dynamics, as e.g. representative for an immune response [26]. Our best model parameterization (**Fig 2H**), for which we focused on the cell-type specific infection dynamics (day 0–7 post infection), led to a reasonable representation of the dynamics for the proportion of infected cells per cell type (**Fig 2I**), as well as viral loads within the observed ranges (**Fig 2J**), by slightly underestimating the dynamics within basal cells, as well as the peak viral load. Based on previous estimates indicating that spread of SARS-CoV-2 relies predominantly on cell-to-cell transmission (~90% of infections [28]), we obtain local infection spread that, in combination with the regenerative capacity of the tissue, determine the oscillatory dynamics (**Fig 2H**, Movie2 at https://github.com/GrawLab/SARS-ALIculture and compare Fig S7 in [12]). Assuming infection spread to be more dominated by cell-free virus transmission, our model would predict higher peak viral loads and more substantial loss of ciliated cells at early time points (**S3 Fig**). Thus, combining data from different sources, our analyses provided a first physiologically relevant spatially-resolved representation of SARS-CoV-2 infection within bronchial epithelial ALI-culture systems accounting for different cell type dynamics.

### Spatial effects and tissue pathology

**Fig 3:**
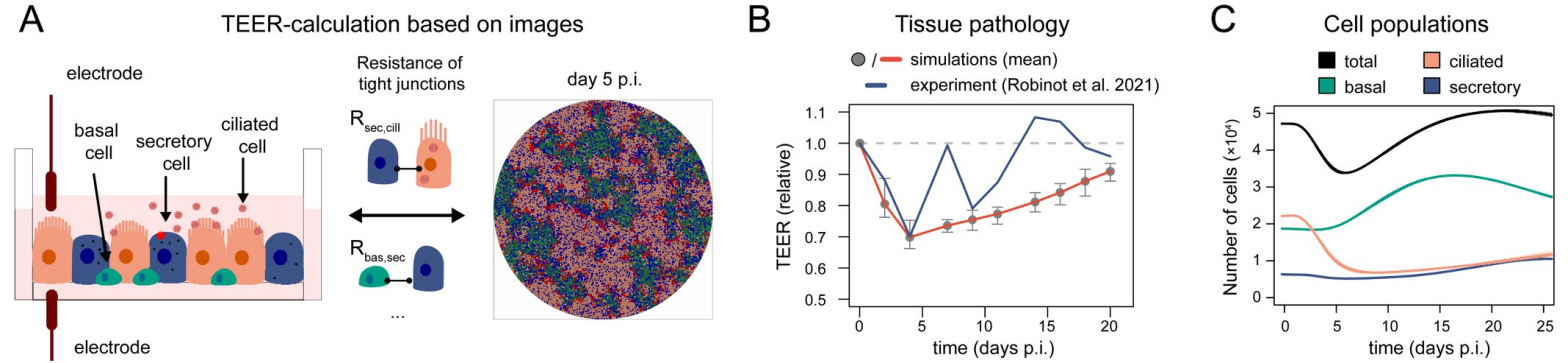
Determining tissue integrity. (**A**) Schematic of relating experimental measurements of transepithelial electrical resistance to image data based on individual cell tight junctions and spatial patterns. (**B**) TEER-dynamics for simulations of bronchial tissue (grey dots/red line) in comparison to previous experimental measurements from the literature (blue line) [12]. Data points indicate the mean (dots/red solid line) and min-max range (whiskers) across 10 independent simulation runs. (**C**) Corresponding dynamics of total and cell type specific cell numbers across the 10 simulations (mean–solid line, range–shaded area). Variations are minor with regard to the population sizes making the ranges hardly visible.

One of the hallmarks defining severity of SARS-CoV-2 infection within patients is the inferred pathology mediated to airway epithelial tissue in the lung [31]. Quantifying tissue pathology and relating it to functional loss, as well as evaluating the regenerative potential, are essential to predict disease progression. Within experimental tissue culture systems, tissue integrity is usually quantified by the transepithelial electrical resistance (TEER), determined by the electrical resistance between electrodes positioned at the apical and basal side of the culture [32]. However, these types of measurement cannot be obtained from patients impairing the ability to relate tissue integrity measures to pathology and possible disease progression. To overcome this issue, we developed a novel method that allows us to quantify tissue integrity based on image data (**Fig 3A**). The method analyses cell type specific membrane connections and relates them to TEER measurements, allowing us to quantify the resistance mediated by the present tissue pattern (see *Materials and Methods* for a detailed description of the method). Using TEER measurements of differentiating bronchial HAE culture systems [21] to parameterize resistance of cell type-specific tight junctions, our simulated culture systems show a rapid loss of tissue integrity after SARS-CoV-2 infection with only 70% (range 66–75%) of TEER remaining at day 4 post infection compared to day 0, comparable to experimental measurements (**Fig 3B**) [12]. The integrity is slowly repaired afterwards reaching almost homeostatic conditions within 25 days after infection, in correspondence to the reconstitution of the individual cell populations during tissue regeneration (**Fig 3C**). However, the inferred regeneration dynamics and oscillations seem to be slower than observed experimentally [12], indicating the possibility for altered cell proliferation and differentiation dynamics during acute tissue damage (**Fig 1** and **Table 1**). In general, we do not observe a direct proportionality between the total number of cells and the tissue integrity measured by TEER, as the particular cell types and their connectivity influence these values.

### Influence of cellular composition on tissue pathology and regeneration dynamics

The replication efficacy of SARS-CoV-2 is observed to vary dependent on the specific respiratory tissue, with nasal epithelium showing the highest permissiveness [33–35]. To analyze the effect of tissue composition and regeneration dynamics on the progression of SARS-CoV-2 infection, we additionally applied our model on epithelial differentiation (**Fig 1A**, Eq (1)) to HAE culture systems of nasal epithelium [36], and bronchial epithelial cells exposed to different concentrations of cigarette smoke extracts (CSE) during development, to determine cellular turnover under these conditions. Hereby, the latter condition simulated impaired epithelial restructuration due to smoke exposure [21,37,38].

**Fig 4:**
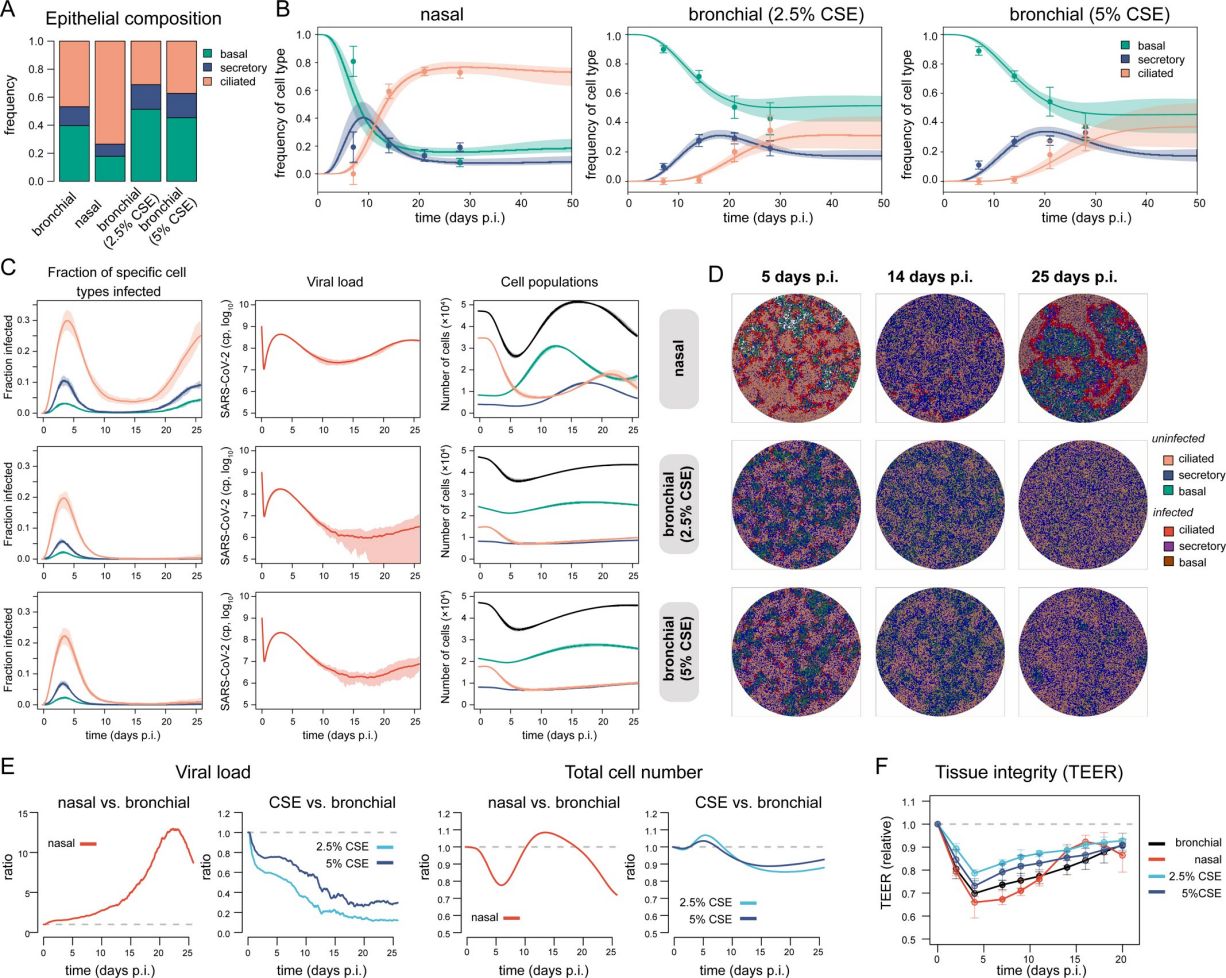
Tissue composition and infection kinetics. (**A**) Composition of bronchial and nasal airway epithelium, including bronchial epithelium exposed to cigarette smoke extract (CSE) at different concentrations [21,36]. (**B**) Relative proportion of each cell type during differentiation of ALI-cultures of the particular epithelium based on previous experimental data (mean/standard deviation = dots/whiskers) [21,36] with model predictions fitting the regeneration model shown in **Fig 1B** to the data. The best fit (solid line) and 90%-prediction intervals for each cell type are shown (basal-green, secretory-blue, ciliated-orange) with parameter estimates given in **Table 3**. (**C**) Simulated dynamics for the relative proportion of specific cell types infected, viral load, and total cell numbers during SARS-CoV-2 infection given different ALI-culture conditions. The mean and range (min-max) over 10 independent simulations for each condition are shown. (**D**) Corresponding snapshots of infection patterns at specific time points (see also Movie 3–5 at https://github.com/GrawLab/SARS-ALIculture). (**E**) Relative size of viral load and total cell population of the different tissue conditions using bronchial epithelium as a baseline. The ratio of the means across 10 simulations each are calculated. (**F**) TEER measurements across 5 (nasal, CSE) and 10 (bronchial) independent simulations for each condition relative to each TEER measurement at day 0.

Compared to bronchial epithelium, nasal epithelium is characterized by a larger fraction of ciliated cells, with basal cells only making up ~18% at steady state compared to ~40% in bronchial tissue (**Fig 4A** compare to **Fig 1 and S3 Table**) [36]. Our analysis indicates that these different steady states are due to faster differentiation rates of basal into secretory, and secretory into ciliated cells, which are estimated to be roughly 2-fold higher in nasal compared to bronchial tissue with all other parameters being comparable (**Tables 1 and 3 and Fig 4B**). In contrast to nasal and healthy bronchial epithelium, airway epithelium of smokers is usually characterized by basal cell hyperplasia [39–41], with ciliated cells being less frequent (**Fig 4A and 4B**). This is also seen in our estimates on the differentiation dynamics of bronchial epithelium exposed to cigarette smoke extracts by slightly increased loss rates for secretory and ciliated cells (**Table 3**).

**Table 3:** Parameter estimated for development and differentiation of HAE systems for different conditions: Parameters were estimated by fitting the model for epithelial differentiation dynamics (Eqs (1)–(2), Fig 1B) to data from ALI-cultures of nasal epithelium [36] and of bronchial epithelial tissue exposed to 2.5% and 5% cigarette smoke extract (CSE)[21]. Values indicate best estimate and 90%-credible intervals (CrI) of estimates using an Approximate Bayesian Computation approach (pyABC, [23]) running for 15 generations with 2000 particles each.

Parameter	Unit	nasal	bronchial	
		best (90%-CrI)	2.5% CSE best (90%-CrI)	5% CSE best (90%-CrI)
Maximal proliferation rate of basal cells, *α*	d^-1^	0.59 (0.42, 0.60)	0.27 (0.16, 0.77)	0.27 (0.15, 0.77)
Differentiation rate basal-secretory, *λ*_*s*_	×10^−2^ d^-1^	18.17 (15.28, 21.91)	8.94 (8.33, 9.57)	8.11 (7.37, 8.83)
Differentiation rate secretory-ciliated, *λ*_*c*_	×10^−2^ d^-1^	28.69 (22.04, 29.99)	13.89 (12.38,15.24)	11.87 (10.21,13.70)
Loss rate of basal cells, *δ*_*b*_	×10^−2^ d^-1^	0.66 (0.10, 2.09)	1.98 (0.12, 2.10)	1.85 (0.14, 2.10)
Loss rate of secretory cells, *δ*_*s*_	×10^−2^ d^-1^	9.98 (6.43, 13.50)	11.39 (7.46, 15.93)	8.53 (4.50, 13.33)
Loss rate of ciliated cells, *δ*_*c*_	×10^−2^ d^-1^	2.28 (2.00, 3.22)	4.11 (2.45, 6.48)	3.01 (2.01, 6.04)
Maximal capacity of ALI-culture, *N*_*max*_	relative to *N*_*0*_ *= 1*	1.33 (1.26, 1.58)	1.45 (1.11, 1.97)	1.39 (1.10, 1.90)

To determine how the different tissue compositions affect infection dynamics, we simulated SARS-CoV-2 infection within these conditions using the same infection parameters as obtained for bronchial epithelium (**Tables 2 and S1**), in combination with the tissue-specific regeneration and differentiation dynamics (**Table 3**). As expected, given the high availability of ciliated cells, infection within nasal epithelium is faster and more severe than in bronchial epithelium, leading to ~1.5-fold higher fraction of infected ciliated cells compared to bronchial epithelium at day 5 post infection, and a loss of ~80% of ciliated cells at 10 days post infection (**Fig 4C,** compare **Fig 2I**). With tissue regeneration being ~2-fold faster than in bronchial epithelium, higher viral load levels are maintained over longer periods of time supporting oscillatory dynamics and regrowth of infection, including continuous tissue reconstruction (**Fig 4D**). In contrast, bronchial epithelium exposed to cigarette smoke extract does not lead to more severe infection patterns with viral loads decreasing faster than in unexposed tissue (**Fig 4C–4E,** compare **Fig 2I, 2J**). However, tissue regeneration is partly impaired under these conditions, showing reduced ability of the tissue to rebuild homeostatic cell numbers after infection compared to untreated bronchial tissue (**Fig 4E**). This is not visible within the progression of the calculated relative transepithelial electrical resistance values, which only show a slightly slower progression rate of TEER normalization for CSE-treated compared to untreated bronchial tissue, while the faster regeneration capacity of nasal epithelium is confirmed (**Fig 4F**). In summary, our analysis indicates how tissue and cell type specific effects could affect SARS-CoV-2 infection dynamics within human airway epithelial tissue.

## Discussion

Understanding the kinetics of SARS-CoV-2 replication and spread within heterogeneous human airway epithelial tissue is essential to determine disease progression and pathology within patients, and to develop efficient treatment strategies. Air-liquid interface cultures have increased our ability to study the molecular and cellular aspects of SARS-CoV-2 infection under different conditions, but deciphering the contribution of various cell types and molecular processes requires integrative approaches that are able to simultaneously analyze various types of measurements. Here, we developed a computational model that in combination with experimental data can be used to analyze infection dynamics within pseudo-stratified airway epithelium. By applying our model to published experimental data from different experiments, we provide a first assessment of cell type specific infection kinetics, and approach the questions of how different cell types, tissue composition and tissue differentiation kinetics contribute to infection and pathology.

While ALI-culture systems of human airway epithelium have been used frequently to study respiratory viruses (reviewed in [42,43]), surprisingly little information could be found on the quantitative assessment of the turnover and differentiation kinetics of the various cells within these systems. The apparent lack of quantitative data for the cell culture systems used severely impairs the ability to reliably parameterize cellular turnover and, thus, tissue composition at different time points. Some of our estimates (e.g. the turnover of secretory cells (*δ*_*s*_, *λ*_*s*_), as well as the maximal proliferation of basal cells (*α*) (**Table 1**)) are in line with previous estimates on epithelial differentiation obtained for mouse tracheal tissue that reported an average lifetime of secretory cells of 14 (±2) days and a rate of basal to secretory cell differentiation of ~2×10^−3^ h^-1^ [20], as well as a doubling time of basal cells in human airway epithelium of 30–35 hours [44]. However, especially for the ciliated cells, our model predicts a roughly 10-times faster loss rate compared to estimates in mice [20,45]. This is compensated by an equally faster differentiation rate which is related to the impaired ability of parameter inference given the available data (**S1 Fig**). Still, our analysis consistently shows that secretory cells have the fastest turnover of the considered cell types [20]. Differences in cell lines (mice/human), as well as the experimental conditions could additionally relate to these discrepancies. In general, we also find that the differentiation dynamics we infer for homeostatic development is too slow to explain the oscillatory dynamics of viral load and tissue integrity observed during experiments. While we observed tissue reconstitution around day 25 post infection (**Fig 3B**), experimental observations show oscillations with periods in the order of 12–20 days [12,15]. This indicates that additional factors mediating increased tissue regeneration during infection could play a role that need to be taken into account [7,46]. Given the importance for understanding disease progression and pathology, more quantitative and time-resolved measurements of ALI-culture differentiation dynamics including cell counts, cell type specific proportions and images of tissue structure will be needed to appropriately quantify cellular turnover in human epithelial tissue and understand disease progression.

With each cell type within respiratory epithelium contributing to the proper functioning of the tissue, determining their role during infection is important to assess their impact on pathology. For SARS-CoV-2, ciliated cells have been identified as one of the major targets of the virus [12,47]. This correlates with the high expression of ACE2 on these cells, which represents the main target receptor for SARS-CoV-2 [48], as well as the increased viral replication observed in nasal epithelium [49], which is characterized by a larger frequency of this cell type. Modelling and analyzing cell type specific infectivity, our analysis also indicates that ciliated cells are more likely to get infected. While the range of parameter estimates on the duration of the viral eclipse phase (~5h [2.9h, 20.0h]) and the lifetime of productively infected cells (~12.9 h [6h, 28h]) corresponds to previous quantifications [7,26,50–52] independent of the specific cell type (**Table 2**), the lower fraction of refractory cells for ciliated cells compared to the other cell types indicates a higher infectability of this cell type. Further data will be needed to clearly determine if this is mainly due to the location of the cells at the apical side of the tissue, their increased cell surface due to the cilia which facilitate viral encounters, or additional molecular factors. In addition, while the relative ratio of parameter estimates for individual cell type infectivity might be informative, actual parameter estimates for the individual infection dynamics should be taken with care (**Table 2**), given the uncertainty associated with their individual identifiability (**S1** and **S2 Figs**), especially also with regard to the dynamics of cellular turnover (**Table 1**).

With regard to their functionality, the loss of ciliated cells is assumed to play a major role during infection and for pathology [12,47]. Robinot et al. [12] reported on dedifferentiation of ciliated cells and loss of cilia during SARS-CoV-2 infection within *ex vivo* and *in vivo* models. Damage of cilia and their impaired functionality could prevent removal of infectious mucous and facilitate spread of infection to deeper lung sections [12]. The preferential targeting of ciliated cells and their dysfunctionality, e.g., impaired synchronicity of cilia beating, accelerated shedding of infected cells, or impaired cell differentiation have already been reported for several other respiratory viruses including respiratory syncytial virus (RSV) [53], influenza [54–56] and rhinoviruses [57]. Accounting for active transport of virions by cilia in our model besides viral diffusion could help to investigate the role of cilia loss for disease progression and pathology, as well as their importance for viral replication [58].

Loss of ciliated cells is also a hallmark of chronic obstructive pulmonary disease (COPD), with smoking one of the major risk factors for its development. Smoking also appears as one of the major risk factors for severe disease progression once SARS-CoV-2 infection is established [35,59] with other studies reporting a reduced infectivity of smokers by SARS-CoV-2, with these controversial findings partly referred to as the smoker paradox [60]. While a reduced frequency of ciliated cells among HAE cells within smokers could explain the impaired ability of SARS-CoV-2 to establish infection, cigarette smoke extract has been found to have a detrimental effect on airway epithelial cell differentiation [21,41], affecting tissue regeneration. Our analyses indicate altered differentiation dynamics within CSE-exposed tissue with slightly increased loss rates of secretory and ciliated cells in comparison to untreated bronchial tissue. Furthermore, using corresponding tissue compositions, we generally observe lower viral loads within simulated CSE-exposed bronchial tissue compared to untreated tissue (**Fig 4E**), relating to the potential impaired ability to establish infection. While simulated CSE-exposed tissues show an impaired ability of the tissue to recover after infection judged by the number of cells (**Fig 4E**), the calculated relative transepithelial electrical resistance does not indicate slower tissue regeneration compared to untreated bronchial tissue (**Fig 4F**). However, our models were simulated assuming the same total number of cells for each condition, neglecting possible reduced cell numbers within CSE-exposed epithelium. With the actual cell number having a substantial impact on absolute TEER values, total cell counts for each HAE culture system would be needed to reliably connect tissue composition and tissue integrity. Our novel approach to calculate tissue integrity from image data by relying on the parameterization of electrical resistance of cell-type specific tight junctions shows its general ability by appropriately predicting early tissue destruction under SARS-CoV-2 infection while being parameterized based on homeostatic conditions (**Fig 3B**) [12,21]. Simultaneous measurements of TEER and cell counts will be needed to reliably parameterize electrical resistance of cell-type specific tight junctions and to improve our method.

One general observation in our attempt to explain data of infection dynamics within HAE culture systems indicated the necessity to consider processes that limit the spread of infection over time, e.g. refractory cell populations (ODE) or inhibition of viral transmission dynamics (CPM), which could be seen as a surrogate for immune effects. Similar observations have been made within other modelling studies [26]. Innate immune responses, especially type I and III interferons, have been found to play an essential role in early viral control, with SARS-CoV-2 having also evolved mechanisms to counteract their efficacy [61,62]. With timing and magnitude of the immune response being critical for establishment and progression of infection [63–66], quantifying cell type specific kinetics of innate immune activation and efficacy will be essential to predict disease progression within different respiratory tissues. In our analyses we assumed that all tissues experience similar immune efficacies in terms of timing and magnitude. With recent studies reporting on different immune transcription profiles between nasal and bronchial epithelium in response to SARS-CoV-2 infection [11], and potential impairments of immunity within smokers, accounting for these differences, as well as modelling immune processes in more detail, will improve comparability of measurements of tissue integrity between conditions (**Fig 4F**) and to determine the role of specific immune responses for the progression of tissue pathology.

Our approach represents a first step towards quantifying cell-type specific infection kinetics and their importance for disease progression and pathology within human airway epithelium during SARS-CoV-2 infection. It provides a methodological framework to disentangle the contribution of different factors, such as tissue heterogeneity, cell-type specific dynamics and local interactions, to disease progression and pathology, as well as an approach to determine tissue integrity from imaging data. Thereby, our approach is able to simultaneously integrate different experimental observations and determine individual cell dynamics without the necessity for in-depth single-cell sequencing analyses. With several modelling environments proposed recently that simulate viral infection dynamics within tissues at different levels of detail [17–19,67,68], our approach represents a first setup for a framework that accounts for a pseudostratified, heterogeneous tissue structure to study tissue integrity under physiologically relevant conditions. By combining information from several studies that rely on diverse experimental protocols [12,21,27,36] our ability to reliably explain all experimental observations is impaired, as for example different culture media and settings could impact cell differentiation dynamics. As cellular turnover and innate immune dynamics were found to be key processes influencing infection progression and pathology, additional data on tissue differentiation (as e.g. time-resolved cell counts and relative cell type frequencies), as well as immune activation kinetics will be needed to provide a systematic and quantitative understanding of the impact of tissue composition on infection dynamic. Using advanced parameter inference tools [23,69] to simultaneously adapt our individual cell-based model to these various measurements will help us to assess and predict the role of different airway epithelial tissues on SARS-CoV-2 infection dynamics.

## Materials & methods

### Air-liquid interface cultures

Nasal and bronchial ALI cultures were purchased from Epithelix (https://www.epithelix.com) and were received when they had been differentiated for 6 weeks and displayed the correct TEER. Once received the samples were placed in fresh media and allowed to recover. Two days post-delivery samples were washed to remove mucus from the apical membrane. The number of cells in an ALI culture was determined by fixing the transwells and staining for DAPI. The cell number was then generated by imaging on a Zeiss CD7 Cell Discoverer and nuclei counting with Illastik (see **S1 Data** and **S4 Fig**).

### Model of cellular turnover and epithelial regeneration

To model the differentiation and cellular turnover within the Air-Liquid Interface culture system we consider the three main cell types of human bronchial epithelium including basal (*B*), secretory (*S*) and ciliated (*C*) cells. Basal cells continuously proliferate at a density-dependent proliferation rate, with a maximal proliferation rate *α*. The cells follow a linear differentiation pathway into secretory and ciliated cells at rate *λ*_*s*_ and *λ*_*c*_, respectively [20]. All cell types die at a corresponding death rate *δ*_*i*_ with *i*∈{*B*,*S*,*C*}. The whole dynamics is then described by the following system of ordinary differential equations.


$$\begin{array}{c} \frac{dB}{dt}=\alpha (1-\frac{N}{N_{max}})B-(\lambda_{s}+\delta_{b})B\\ \frac{dS}{dt}=\lambda_{s}B-\lambda_{c}S-\delta_{s}S\\ \frac{dC}{dt}=\lambda_{c}S-\delta_{c}C \end{array}$$ (1)

Hereby, *N* = *B*+*S*+*C* defines the total cell concentration and *N*_*max*_ the maximal carrying capacity of the culture system. To account for a Γ-distributed differentiation process, the system in Eq (1) was then extended, assuming that basal and secretory cells pass through *k+1* intermediate steps before becoming a secretory or ciliated cell, respectively. The standard model for cell regeneration and differentiation is then defined by

$$\begin{array}{c} \frac{dB_{0}}{dt}=\alpha (1-\frac{N}{N_{max}})(\sum_{i=0}^{k}B_{i})-({k\lambda }_{s}+\delta_{b})B_{0}\\ \frac{dB_{i}}{dt}=k\lambda_{s}{(B}_{i-1}-B_{i})-\delta_{b}B_{i},i=1,\ldots ,k\\ \frac{dS_{0}}{dt}={k\lambda }_{s}B_{k}-{k\lambda }_{c}S_{0}-\delta_{s}S_{0}\\ \frac{dS_{i}}{dt}=k\lambda_{c}{(S}_{i-1}-S_{i})-\delta_{s}S_{i},i=1,\ldots ,k\\ \frac{dC}{dt}=\lambda_{c}S_{k}-\delta_{c}C \end{array}$$ (2)

with the total number of cells, *N*, calculated accordingly, i.e., *N* = *B*_0_+*B*_1_+⋯. For the analysis and parameter estimation, the number of intermediate steps was set to k = 4, i.e., having 5 different compartments (0,1,..,4), because it fits the data reasonably well and is a tradeoff between a non-exponential distribution (*k* = 1) and a computational low cost model to allow for reasonable distributions of differentiation durations.

### Model of cell-type specific infectivity and viral dynamics

To model the infection dynamics of SARS-CoV-2 within the human epithelial cultures, the standard model for cell regeneration and differentiation (Eq (2)), is extended by the infection dynamics for each of the different cell populations. Basal, secretory and ciliated cells get infected proportional to the virus concentration *V* at cell-type specific infection rates *β*. After a viral eclipse phase with an average duration of 1/*κ*, infected cells *I* will turn into productively infected cells *J* and start exporting virus at rate *ρ*, having an average productive lifetime of 1/*δ*_*I*_. For simplicity and given the fast turnover of infected cells, we neglect the loss of non-productively infected cells due to cell death. Each of the different rates of transmission, cell death, eclipse phase and viral production are initially considered to be cell-type specific. The additional equations to Eqs (1)/(2) (without accounting for the compartmentalization in the notation) are then given by

$$\begin{array}{c} \frac{dB}{dt}=\alpha (1-\frac{N}{N_{max}})B-(\lambda_{s}+\delta_{b})B-\beta_{b}VB\\ \frac{dB_{I}}{dt}=\beta_{b}VB-\kappa_{b}B_{I}\\ \frac{dB_{J}}{dt}=\kappa_{b}B_{I}{-\delta }_{Ib}B_{J}\\ \frac{dS}{dt}=\lambda_{s}B-\lambda_{c}S-\delta_{s}S-\beta_{s}VS\\ \frac{dS_{I}}{dt}=\beta_{s}VS-\kappa_{s}S_{I}\\ \frac{dS_{J}}{dt}=\kappa_{s}S_{I}{-\delta }_{Is}S_{J}\\ \frac{dC}{dt}=\lambda_{c}S-\delta_{c}C-\beta_{c}VC\\ \frac{dC_{I}}{dt}=\beta_{c}VC-\kappa_{c}C_{I}\\ \frac{dC_{J}}{dt}=\kappa_{c}C_{I}{-\delta }_{Ic}C_{J}\\ \frac{dV}{dt}=\rho_{b}B_{J}+\rho_{s}S_{J}{+\rho }_{c}C_{J}-cV \end{array}$$ (3)

Hereby, the parameter *c* defines the viral clearance rate. In addition, *N* is now defined by $N=B+B_{I}+B_{J}+S+S_{I}+S_{J}+C+C_{I}{+C}_{J}$, i.e., determining the total concentration of cells within the system.

Analogously to cell differentiation, we also used compartmentalization for the viral eclipse phase, 1/*κ*, and the average lifetime of productively infected cells, 1/*δ*_*I*_, with *k* = 4 intermediate steps each used for parameter fitting.

With this standard model for infection (Eq (3)) not able to explain the data (see main text), we extended the model by additionally considering a proportion of cells that is refractory to infection, *X*_*R*_, *X*∈{*B*,*S*,*C*} for each of the three cell types considered. These populations account for the spatial separation of cells within the culture and within different layers (basal vs. secretory/ciliated), and could also be interpreted as cell protected by innate immune mechanisms [26]. The corresponding equations in Eq (3) above are then modified and extended by

$$\begin{array}{c} \frac{dB}{dt}=(1-f_{b})\alpha (1-\frac{N}{N_{max}})(B+B_{R})-(\lambda_{s}+\delta_{b})B-\beta_{b}VB\\ \frac{{dB}_{R}}{dt}=f_{b}\alpha (1-\frac{N}{N_{max}})(B+B_{R})-(\lambda_{s}+\delta_{b})B_{R}\\ \frac{dS}{dt}={(1-f_{s})\lambda }_{s}(B+B_{R})-\lambda_{c}S-\delta_{s}S-\beta_{s}VS\\ \frac{dS_{R}}{dt}={f_{s}\lambda }_{s}(B+B_{R})-\lambda_{c}S_{R}-\delta_{s}S_{R}\\ \frac{dC}{dt}={{(1-f}_{c})\lambda }_{c}(S+S_{R})-\delta_{c}C-\beta_{c}VC\\ \frac{dC_{R}}{dt}={f_{c}\lambda }_{c}(S+S_{R})-\delta_{c}C_{R} \end{array}$$ (4)

with *f*_*X*_, *X*∈{*B*,*S*,*C*}, denoting the fractions of the corresponding cell types that become refractory, and $N=B+B_{R}+B_{I}+B_{J}+S+S_{R}+S_{I}+S_{J}+C+C_{R}+C_{I}{+C}_{J}$ throughout. Please note that the compartmentalization for the processes of cell differentiation, the viral eclipse phase, and the lifetime of productively infected cells (analogously to Eq (2)) were applied as well, with Eq (4) then representing the model that best explained the experimental data.

### Parameter estimation for cellular turnover and epithelial regeneration

We fitted the model shown in Eq (2) to the data of Schamberger et al. [21] (and de Borja Callejas et al. [36]), using an Approximate Bayesian Computation-Sequential Monte Carlo (ABC-SMC) method [23]. The model was fitted to the data that were obtained by digitalizing the corresponding figures considering the experimentally observed errors with an additional 2% Gaussian noise to correct for data points where errors could not be observed. In addition to the cellular frequencies, we also included a penalty term that ensured that absolute cell numbers maintained reasonable values when fitting the relative frequencies. As ALI-cultures reach confluence and full differentiated epithelium in between 28–50 days after seeding (e.g. https://www.epithelix.com/products/mucilair and https://www.stemcell.com/), additional penalty terms were added to ensure that the individual cell populations reached steady state at around day ~45–50. The cost function for each cell type is then defined by

$${RSS}_{X}=\sum_{t_{i}}{\left(\frac{\mu_{exp}(t_{i})-\mu_{sim}(t_{i})}{\sigma_{exp}(t_{i})}\right)}^{2}+\frac{1}{5}\sum_{t=46}^{51}{\left(\frac{\mu_{sim}(t)-\mu_{sim}(45)}{w_{1}}\right)}^{2}+\frac{1}{10}\sum_{t=51}^{61}{\left(\frac{\mu_{sim}(t)-\mu_{sim}(50)}{w_{2}}\right)}^{2}$$

with *t*_*i*_ = 7, 14, 21, and 28 days defining the time points of measurements with actual observations, and *μ*_*sim*_(*t*), *μ*_*exp*_(*t*) and *σ*_*exp*_(*t*) the predicted and measured mean and standard errors of the particular cell type, respectively. The parameters *w*_1_ and *w*_2_ define weights to enforce the system to reach a steady state at ~45 days. Here, we set *w*_1_ = 0.025 and *w*_2_ = 0.005 to ensure a smooth transition from the dynamics inferred from the experimental measurements (first term) towards the assumed steady state of the system based on qualitative assessments in the absence of comparable experimental data. The choice of the weights will impact the transition dynamics and, thus, the estimated model parameters: Larger values of *w*_1_and *w*_2_ will delay the model to reach steady state.

The complete cost function is then given by

$$RSS=\sum_{X\in \{B,S,C\}}{RSS}_{X}+{\left(\frac{0.95-N(45)}{w_{3}}\right)}^{2}$$

The second term comprises the penalty for the total cell count, with *N*(45) giving the total relative cell count at day 45, and *w*_3_ the weight set to *w*_3_ = 0.2.

The cost function was minimized by Approximate Bayesian Computation (pyABC, [23]), using 5000 particles for initial calibration and 2000 particles per generation. Each evaluation run for up to 15 generations until sufficient convergence was reached. As expected, given the complexity of the model and the available data, correlation analysis indicates that not all parameters are identifiable, with especially the cell proliferation rate α and the maximal cell number *N*_*max*_ experiencing strong correlations (**S1 Fig**). Prior distributions for individual parameters were defined by α~*U*(0.1, 0.6), *λ*_*s*_~*U*(0.05, 0.3), *λ*_*c*_~*U*(0.05, 0.3), *δ*_*b*_~*U*(0.001, 0.021), *δ*_*s*_~*U*(0.02, 0.16), and *δ*_*c*_~*U*(0.02, 0.07) with all units given per day.

### Parameter estimation to determine cell-type specific infectivity

Parameters describing cell-type specific infectivity were estimated by fitting the model shown in Eqs (3/4) to the data obtained from Ravindra et al. [27]. These data contained information from scRNAseq on the frequency of infected basal, secretory and ciliated cells at 1, 2 and 3 days post infection of a bronchial HAE system with SARS-CoV-2.

The model was fitted to the data by Approximate Bayesian Computation (pyABC, [23]), running for 30 generations with 1000 calibration particles and 1000 particles per generation. The previously determined best estimates for bronchial epithelium were considered for epithelial cell turnover (**Table 1**). According to the measurements from Ravindra et al. at 1h post infection [27], an initial infection dose of *V*_0_ = 2.8×10^6^ RNA copies was used.

### A cellular Potts model of the ALI-culture system

We used the cellular Potts modelling framework within the software tool Morpheus [22] to simulate the spatio-temporal dynamics of infection and tissue regeneration within the pseudo-stratified epithelium of the HAE cultures system. The cellular Potts model is a grid-based, individual cell-based model which allows to simultaneously account for inter- and intracellular dynamics across multiple scales. Each cell consists of several grid sites and aims at reaching a specified target volume and perimeter. Heterogeneous cell morphologies are reached as a cell aims at minimizing its global energy, which is defined by its surface tensions and characterized by the so-called Hamiltonian [16,22]. The CPM implementation with its flexible cell shape allows us to account for varying cell volumes with increasing cell densities. This is determined by the parameters volume strength and the so-called aspherity, *V*_*S*_ and Ψ, which define the strength with which a cell tries to reach/maintain the desired target volume and surface area, respectively, and penalizes any deviations from them (see Morpheus and the documentation therein for a detailed explanation of the implementation [22]).

The model distinguishes between basal, secretory and ciliated cells. To simulate a pseudostratified epithelium with basal cells located at the basal membrane of the culture, *V*_*S*_ for basal cells is only 1/10 of the corresponding value for secretory and ciliated cells (**S1 Table**). With this parameterization, basal cells are “compressed” as secretory and ciliated cells increase in numbers, simulating the “overgrowth” of basal cells by the other cell types (see Movie1 at https://github.com/GrawLab/SARS-ALIculture).

### Cell proliferation, differentiation and turnover

The dynamics of the individual cell types with regard to cell proliferation, differentiation and cellular turnover follows the individual processes already defined within Eqs (1/2) and Eqs (3/4) (see also **Figs 1B** and **2A**). Parameterizations are based on the previously estimated parameters (**Table 1**) with stochastic simulations accounting for cellular heterogeneity. To this end, the different continuous rates were translated into probabilities for the discrete events within the CPM dependent on the used timestep size assuming a constant hazard model. For example, with *δ*_*S*_ defining the loss rate of secretory cells within the ODE-model, the probability of a secretory cell to die (i.e., to change compartments according to Eq (2) to account for a Γ-distributed lifetime) within a time step Δ*t* in the CPM is calculated by $p(\Delta t)=1-e^{-\delta_{S}\Delta t}$. Upon cell death, dead cells are removed immediately, i.e., not inhibiting cell proliferation dynamics or the calculation of the transepithelial electrical resistance (TEER) (see below). Due to the dynamic cell morphologies within the CPM and to account for the pseudostratified tissue when modelling density dependent cell growth, proliferation of basal cells is influenced by the total number of cells within the cell culture, and not solely the local surrounding showing reasonable tissue dynamics and growth kinetics.

#### Cell-free infection

Cell-free infection is simulated by additionally accounting for the extracellular virus concentration, *V*. Individual cells get infected dependent on the cell type specific transmission parameter *β*_*X*_, *X*∈{*B*,*S*,*C*} and the total viral concentration at their cellular surface. Once turning productive, productively infected cells will constantly produce virus contributing to the extracellular viral concentration at the specific grid site. Viral concentration is modelled as a field which diffuses through the system at a diffusion rate *D*_*V*_ = 60 μm^2^/h comparable to previous effective diffusion rates used [70].

#### Cell-to-cell transmission

In addition to infection by cell-free virus, we also allow for direct cell-to-cell transmission between productively infected and uninfected cells. The probability at which cells get infected depends on the type of the uninfected cells (*X*∈{*B*,*S*,*C*}), characterized by the relative infection parameter *β*_*cc*,*X*_, as well as the cell-types of the surrounding productively infected and, thus, potentially infecting cells, characterized by the relative infecting parameter *ρ*_*cc*,*X*_, i.e., analogous to the transmission and viral production parameters for cell-free infection. Without specific knowledge or data on cell-type-specific effects on cell-to-cell transmission, we used the relative relationships of the estimated cell-type specific viral transmission and production rates from the ODE model (**Table 2**) to parameterize *β*_*cc*,*X*_ and *ρ*_*cc*,*X*_. We arbitrarily set *β*_*cc*,*b*_ = 1 and *ρ*_*cc*,*b*_ = 1, considering basal cells as a baseline, and determined the other values based on the ratio of *β*_*s*_/*β*_*b*_, *β*_*c*_/*β*_*b*_ and *ρ*_*s*_/*ρ*_*b*_, *ρ*_*s*_/*ρ*_*b*_, respectively (see **Table 2**). The probability of an uninfected basal cell to get infected is then determined by

$$P_{cc,b}=\beta_{cc,b}(N_{j,b}\rho_{cc,b}+N_{j,s}\rho_{cc,s}+N_{j,c}\rho_{cc,c})$$

with *N*_*j*,*b*_, *N*_*j*,*s*_ and *N*_*j*,*c*_ defining the number of productively infected basal, secretory and ciliated cells, respectively, which are in contact to the corresponding cell. For the other cell types, the probability is calculated analogously. Please note that for basal and secretory cells, the probability to get infected had to be additionally reduced by a factor of 0.2 to further separate their infection dynamics from those of ciliated cells, indicating a clearly higher potential of ciliated cells to get infected.

#### Adaptation of infection dynamics

To further adapt the infection dynamics within our spatially explicit CPM simulation, we additionally control the effectiveness of infection by two weighting factors *w*_*cf*_ and *w*_*cc*_, which put a weight on the previously determined values of cell-free viral transmission, *β*_*X*_, and cell-to-cell transmission, *β*_*cc*,*X*_, respectively, with *X*∈{*B*,*S*,*C*}. By changing the weighting factor while keeping all other parameter relationships constant, we are able to control the initial speed of infection within our spatial simulation and adapt our simulation to the observed dynamics by only changing a limited number of parameters. For cell-free viral transmission, *w*_*cf*_ translates the transmission rates obtained from the ODE approach to the effective area of the viral concentration in the spatially-explicit CPM, corresponding to the cellularization method introduced in [71]. In addition, both transmission parameters are further controlled by the relative contribution of cell-free and cell-to-cell transmission to infection determined by *f*_*cc*_. Thus, for each specific cell type *X*∈{*B*,*S*,*C*}, the transmission rates are then defined by ${\tilde{\beta }}_{X}=(1-f_{cc})w_{cf}\beta_{X}$ and ${\tilde{\beta }}_{cc,X}=f_{cc}w_{cc}\beta_{cc,X}$, respectively.

#### Innate immune protection

As a model extension to additionally explain the observed infection dynamics and mimicking the refractory cell populations considered within the ODE-model [26], we additionally considered the effect of an immune response within our simulations. Reflecting innate immune responses, immune protection develops in response to the pathological damage that is caused by the infection, and would reduce cell-free and cell-to-cell transmission, i.e., *β*_*cc*,*X*_ and *β*_*cc*,*X*_, respectively with *X*∈{*B*,*S*,*C*}. We specifically considered the damage within the apical cell layer comprising ciliated and secretory cells to control these dynamics, i.e., with increasing damage leading to a saturation of the transmission dynamics. Immune protection, *E*(*t*), is then calculated by

$$E(t)=1-\frac{1}{g_{max}}\left(1-\frac{N_{apical}(t)}{N_{apical,0}}\right)$$

where *N*_*apical*,0_ = *S*+*C* defines the number of ciliated and secretory cells at the start, $N_{apical}(t)=S(t)+S_{I}(t)+S_{J}(t)+C(t)+C_{I}(t)+C_{J}(t)$ the number of uninfected, infected and infectious ciliated and secretory cells at the current time point, and *g*_*max*_ the maximal fraction of the apical layer that could be damaged. We used a default value of *g*_*max*_ = 0.75. Considering immune protection, the transmission parameters are then updated according to ${\tilde{\beta }}_{X}=(1-f_{cc})E(t)w_{cf}\beta_{X}$ and ${\tilde{\beta }}_{cc,X}=f_{cc}E(t)w_{cc}\beta_{cc,X},X\in \{B,S,C\}$. Please note that for analyzing SARS-CoV-2 infection dynamics in nasal epithelium and bronchial epithelium exposed to cigarette smoke extract (**Fig 4**), we used the same kinetics and efficacy of innate immune protection as inferred from bronchial tissue, meaning the same functional values of *E*(*t*) as used in normal bronchial tissue, to restrict possible variations to the differences in the composition and regeneration dynamics of the epithelium. To this end, we determined the mean dynamics of *E*(*t*) across the 10 simulations of bronchial epithelium and loaded them as a function into the other simulations.

#### Default parameterization

As default parameterization, we assume a radial cell culture system with a diameter of *d* = 1.217 *mm* comprising a total of ~4.7×10^4^ cells according to our experimental measurements (**S4 Fig**) and using a spatial resolution of 1 pixel = 1 *μm*. Simulations were performed with a time step size of 0.05 days to simulate the ALI-culture regeneration model for a total of 50 days (**Fig 1**), and a time step of 15 minutes to simulate the infection dynamics for a total time period of 25 days (**Fig 2**). For simulating infection, cell types are distributed according to the assumed steady-state conditions, and infection is introduced after a burn-in phase of 25 time steps. The initial infection dose *V*_*0*_ was calculated to obtain the same number of initially infected cells as in the ODE system that used an initial infection dose of 2.6 × 10^6^ RNA copies (obtained from [27], viral load at 1h). Considering the size of the simulated radial cell culture with radius *r* and the total number of cells *N*, as well as the local force of infection regulated by the weighting factor *w*_*cf*_, the initial viral dose is then given by $V_{0}=\frac{2.8\times 10^{6}}{(\pi /4{)r}^{2}}N\frac{1}{w_{cf}}$. The initial viral load was homogeneously distributed throughout the grid. The xml-code of the model is provided at https://github.com/GrawLab/SARS-ALIculture with all standard parameterizations for the specific CPM parameters also provided in **Tables 1, 2,** and **S1**.

#### Parameter adaptation

A combination of different parameter sweeps was used to improve adaptation of the CPM-model to the experimental data. To limit the number of parameters to vary given the complexity of the model, we concentrated our analysis on the adaptation of the weight factor *w*_*cf*_, as well as an additional weighting factor *w*_*V*_ that regulated the initial viral infection dose, and, thus, the initial speed of infection. With these limited regulations, it was possible to obtain an appropriate parameterization of the cell-type specific infection dynamics observed in the experimental data [27] in combination with the amount of tissue pathology observed in such culture conditions.

### Estimating transepithelial electrical resistance (TEER) based on image data

To measure tissue pathology within the ALI-culture system we quantified transepithelial electrical resistance (TEER) in our simulations, which is a standard method to measure the integrity of a tissue [32]. It determines the electrical resistance of a tissue layer placed on a semi-permeable membrane by measuring the resistance of the cell layer between electrodes placed at the apical and basolateral compartments of the ALI-culture system. Assuming that the resistance per area of the cellular membranes is much larger than the resistance at the tight junctions (TJ) of the cells, *R*_*membrane*_≫*R*_*TJ*_, the total resistance of the tissue, *R*_*Tissue*_, is the harmonic mean of the resistance of the tight junctions, since they are connected in a parallel circuit. If all tight junctions have equal resistance, this would give us:

$$R_{Tissue}\propto \frac{1}{M_{TJ}}$$

with *M*_*TJ*_ denoting the area covered by the tight junctions. The reported TEER-value is then defined by *TEER* = *R*_*Tissue*_×*M*_*total*_ in Ω×*cm*^2^, with *M*_*total*_ being the total area of the tissue, i.e., *M*_*total*_ = *M*_*TJ*_+*M*_*cells*_ (see also [32]).

With three cell types (basal, secretory, ciliated) within the culture system, as well as uncovered area (medium), the total resistance of the tissue is then given by

$$\frac{1}{R_{Tissue}}=\sum_{i}\frac{1}{R_{i}}=N_{bas,bas}\frac{1}{R_{bas,bas}}+N_{bas,sec}\frac{1}{R_{bas,sec}}+N_{bas,cil}\frac{1}{R_{bas,cil}}+N_{sec,sec}\frac{1}{R_{sec,sec}}{+N}_{sec,cil}\frac{1}{R_{sec,cil}}+N_{cil,cil}\frac{1}{R_{cil,cil}}+N_{empty}\frac{1}{R_{medium}}$$ (5)

accounting for all possible tight junctions for each of the different cell types with each other and the corresponding area covered, with *i* denoting the number of all pixels. Hereby, *R*_*bas*,*bas*_, *R*_*cil*,*cil*_, etc. define the resistance of the particular tight junctions, and *N*_*bas*,*bas*_, *N*_*bas*,*sec*_, etc. the number of pixels with the corresponding connections. To determine the area of each tight-junction combination, we used the images generated by our CPM simulations. For each pixel characterizing a tight-junction (i.e., shown in black), we determined the type of pixels in the direct neighborhood (3×3 area) to classify the type of tight junction dependent on the type of cells in the surrounding. For example, if all pixels belong to either ciliated cells or represent other tight junctions, this would count towards a *cil*-*cil* area. Using a resolution of 2000×2000 pixels for our images (1 image pixel = 0.6×0.6 *μm*^2^), the borders of the tight junctions are usually 1–2 pixels in width.

To parameterize transepithelial resistance values in our simulations, we used TEER measurements for the differentiation of bronchial epithelium under homeostatic conditions ([21], **Fig 1C**). For simplification, we assumed that the cell types within the different layers of the pseudo-stratified epithelium (*basal* vs. *secretory/ciliated*) behave similar:

$$R_{sec,sec}=R_{sec,cil}=R_{cil,cil}$$

$$R_{bas,sec}=R_{bas,cil}$$

This assumption leaves *R*_*bas*,*bas*_, *R*_*bas*,*cil*_, *R*_*sec*,*cil*_ and *R*_*medium*_ as unknown parameters that need to be determined. In addition, a factor accounting for the influence of infectivity of one of the cells to lower the resistance of the particular tight junction was determined as well. Parameters were obtained by adapting the calculation of the simulated culture systems based on Eq (5) to the TEER measurements of untreated bronchial epithelium under homeostatic conditions [21].

## Supporting information

S1 Fig Parameter dependencies. Relative dependencies of estimated regeneration and differentiation parameters using the 10%-best parameter combinations after 15 generations of ABC when fitting the model (Fig 1B, Eq. (1–2)) to the experimental data [21] (Table 1). Numbers in the upper diagonal matrix determine the calculated Pearson correlation coefficient.
(PDF)

S2 Fig Parameter dependencies. Relative dependencies of estimated infection parameters using the 10%-best parameter combinations after 30 generations of ABC when fitting the infection model (Eq (3)) to the experimental data [27] (Table 3). Numbers in the upper diagonal matrix determine the calculated Pearson correlation coefficient.
(PDF)

S3 Fig Influence of viral transmission modes on infection dynamics. (A) Effect of different assumptions for the relative contribution of cell-to-cell transmission, *f_cc_*, to the infection dynamics simulating spread of SARS-CoV-2 within bronchial epithelium. All other parameters were kept the same. Simulated dynamics for the relative proportion of specific cell types infected, viral load, and total cell numbers during SARS-CoV-2 with the mean and range (min-max) over 10 independent simulations for each assumed value of *f_cc_* are shown. (B-C) Relative size of viral load (B) and total cell population (C) of the different assumptions for *f_cc_* using *f_cc_* = 0.9 as a baseline. The ratio of the means across 10 simulations for each condition are calculated.
(PDF)

S4 Fig Cell counts for ALI culture systems. Determined absolute number of cells by DAPI staining within ALI cultures of bronchial and nasal human airway epithelium (see *Materials & Methods*). The mean ±*1.96×*.SE over three independent cultures for each condition are shown (**S1 Data**).
(PDF)

S1 Table Parameters for the cellular Potts model (CPM) of the ALI-culture system. General parameters for initializing the cellular Potts model with specificities for the different cell types. The xml-files for the standard model for cell differentiation and turnover with the infection dynamics within bronchial tissue, including all baseline parameterizations and implemented in Morpheus 2.2.6, is provided at https://github.com/GrawLab/SARS-ALIculture.
(PDF)

S2 Table Comparison of ODE and CPM approach. Comparison of the different biological processes/aspects considered within the original and extended ODE approach, as well as the spatially resolved CPM.
(PDF)

S3 Table Tissue composition at steady-state for different ALI-culture systems. Composition of tissues at steady-state using the best estimates for the differentiation dynamics inferred from the experimental data [21] (see also **Fig 4A/4B)**. Values are in % of total cell number.
(PDF)

S1 Data DAPI cell count data. The original cell count data for ALI-cultures of bronchial and nasal epithelium based on DAPI staining used in S3 Fig.
(XLSX)
