# Supplementary figures and images for: Influence of cell type specific infectivity and tissue composition on SARS-CoV-2 infection dynamics within human airway epithelium
Source: PLoS Comput Biol. 2023 Aug 11;19(8):e1011356. doi: 10.1371/journal.pcbi.1011356 (PMC10446191; doi:10.1371/journal.pcbi.1011356)

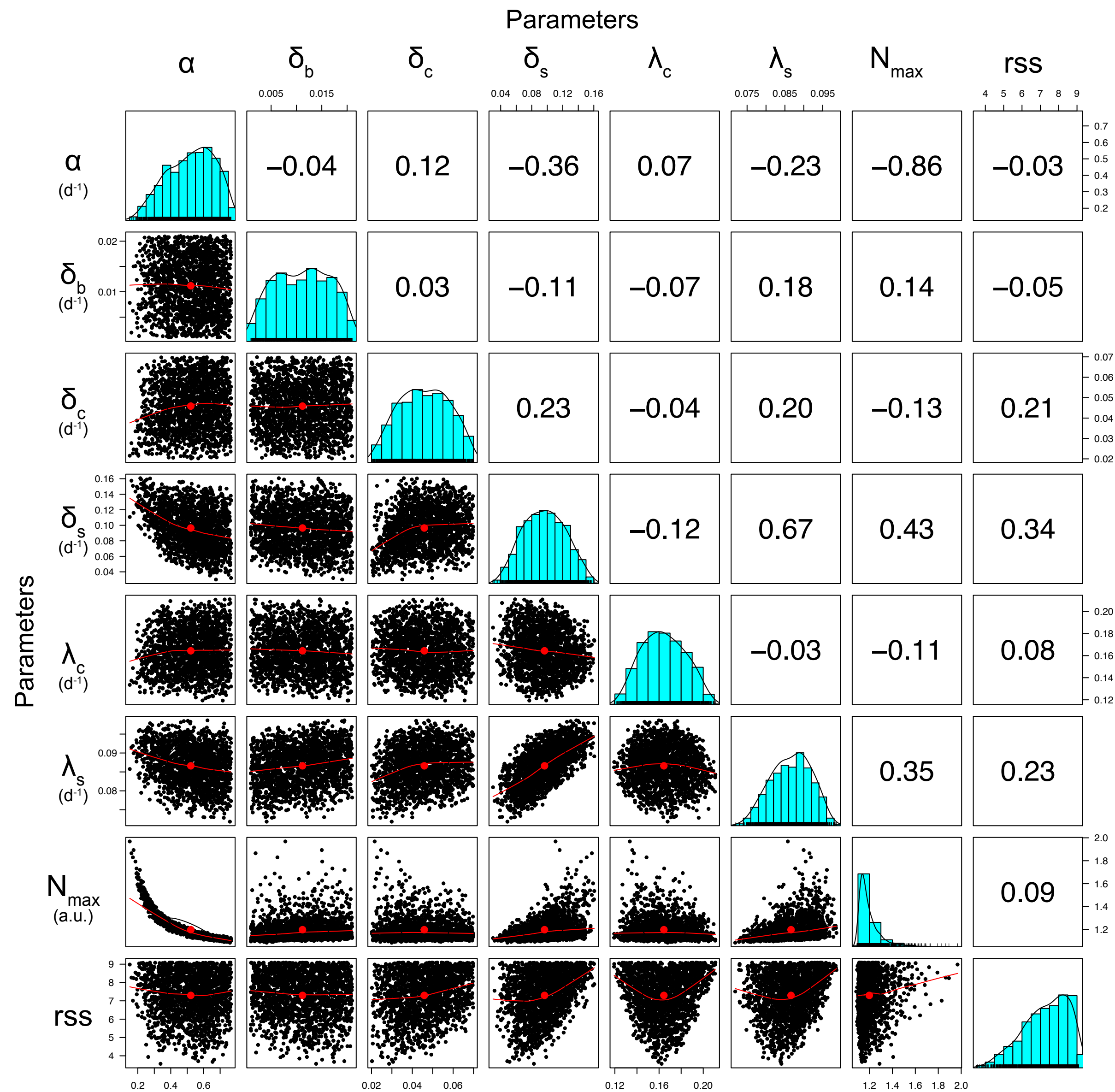

Supplement: S1 Fig — Relative dependencies of estimated regeneration and differentiation parameters using the 10%-best parameter combinations after 15 generations of ABC when fitting the model (Fig 1B, Eq. (1–2)) to the experimental data [21] (Table 1). Numbers in the upper diagonal matrix determine the calculated Pearson correlation coefficient. (PDF) [file pcbi.1011356.s001.pdf]

# Parameters

Parameters

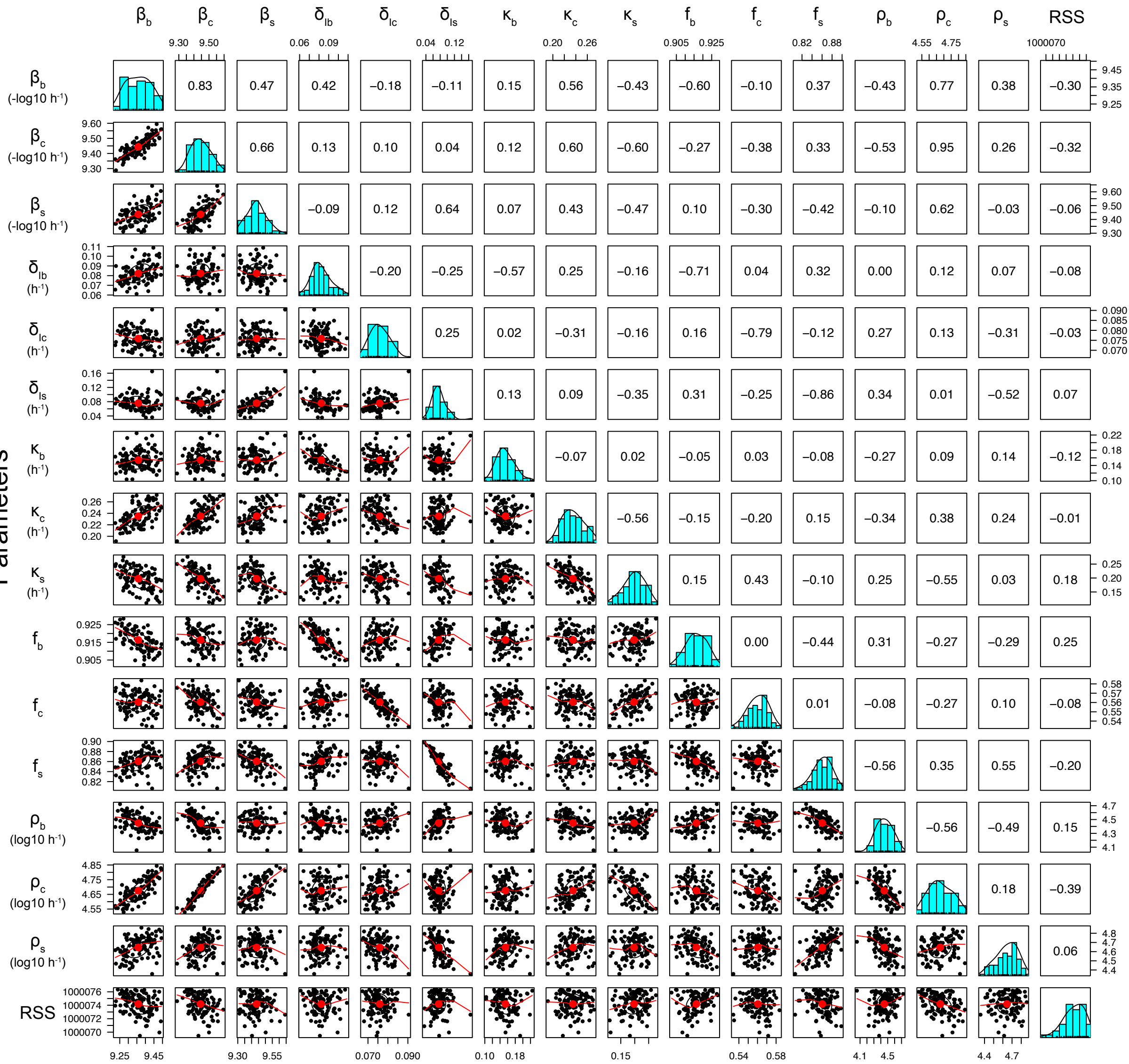

Supplement: S2 Fig — Relative dependencies of estimated infection parameters using the 10%-best parameter combinations after 30 generations of ABC when fitting the infection model (Eq (3)) to the experimental data [27] (Table 3). Numbers in the upper diagonal matrix determine the calculated Pearson correlation coefficient. (PDF) [file pcbi.1011356.s002.pdf]

**A**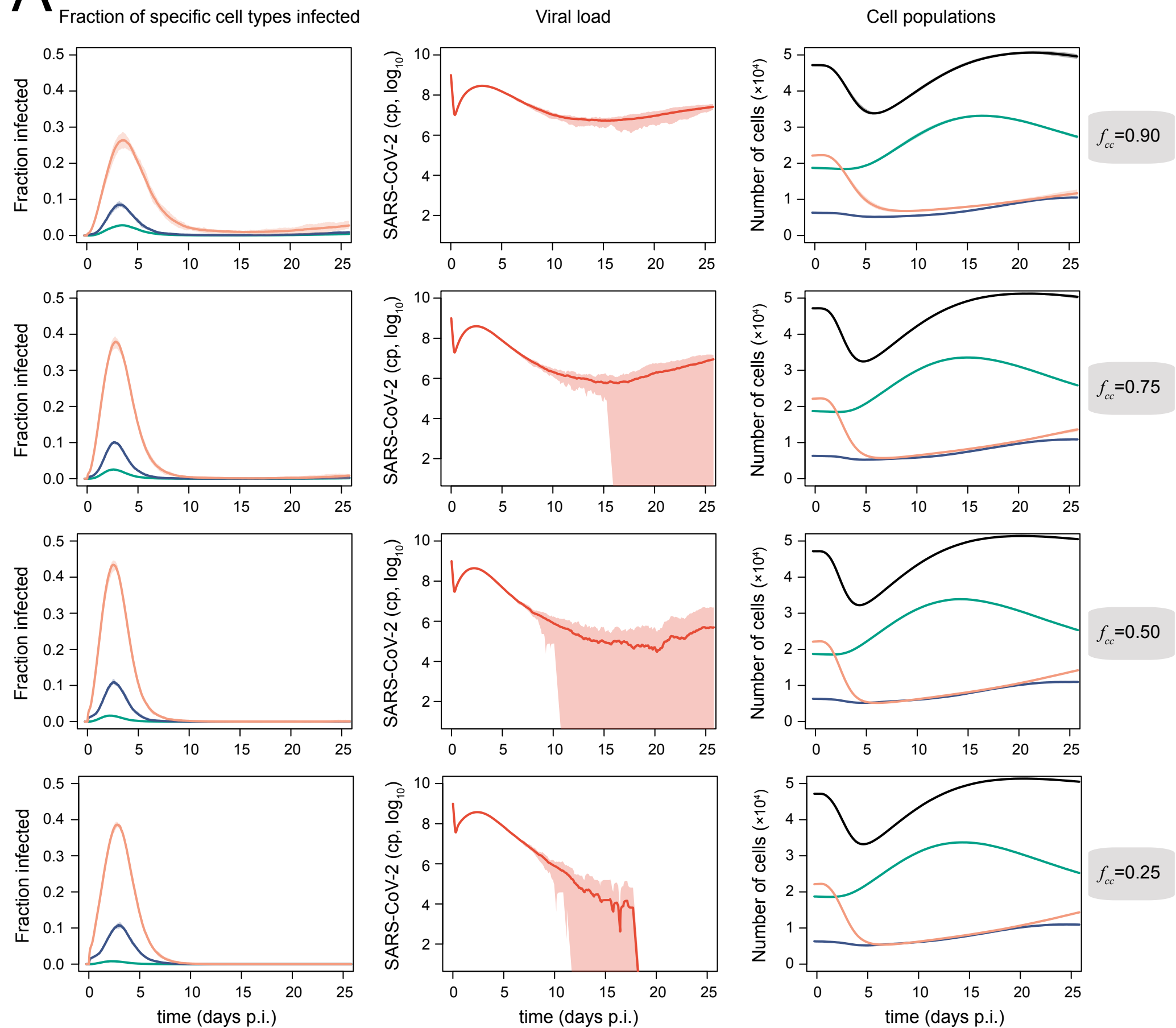**B**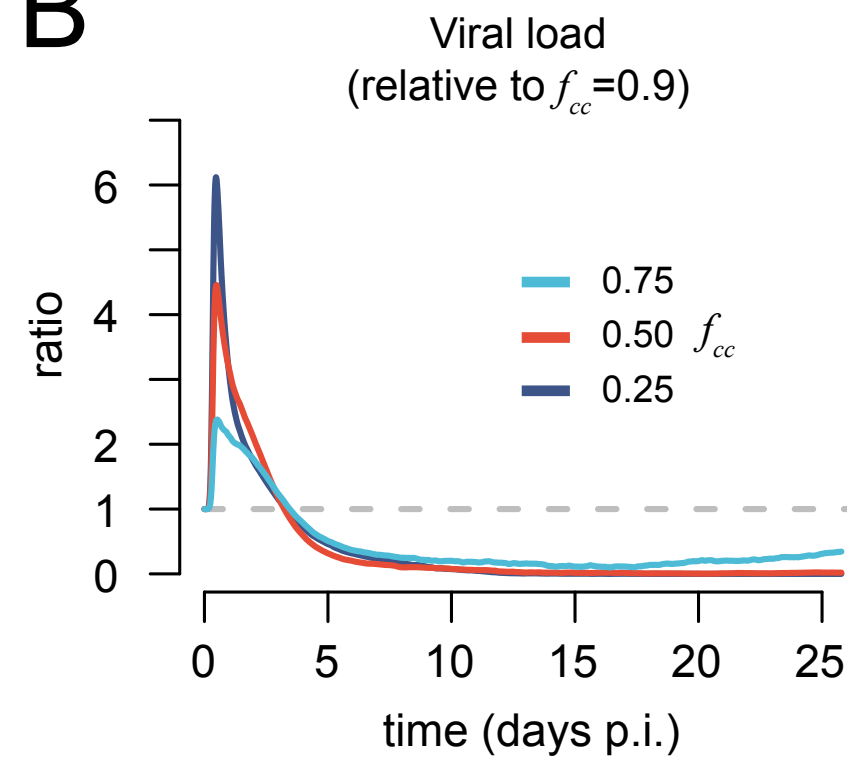**C**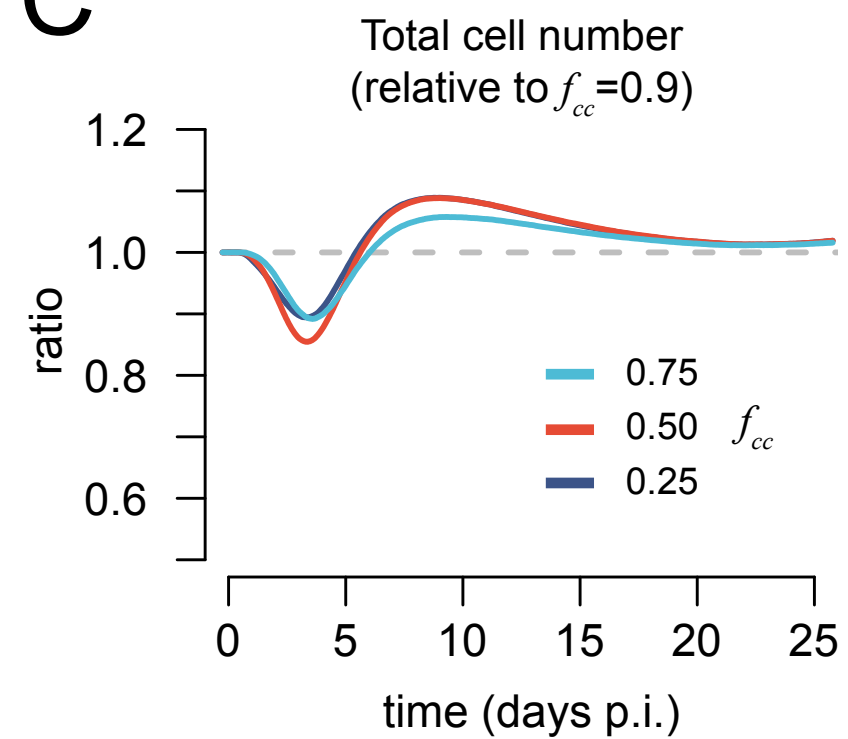

Supplement: S3 Fig — (A) Effect of different assumptions for the relative contribution of cell-to-cell transmission, fcc, to the infection dynamics simulating spread of SARS-CoV-2 within bronchial epithelium. All other parameters were kept the same. Simulated dynamics for the relative proportion of specific cell types infected, viral load, and total cell numbers during SARS-CoV-2 with the mean and range (min-max) over 10 independent simulations for each assumed value of fcc are shown. (B-C) Relative size of viral load (B) and total cell population (C) of the different assumptions for fcc using fcc = 0.9 as a baseline. The ratio of the means across 10 simulations for each condition are calculated. (PDF) [file pcbi.1011356.s003.pdf]

# Cell count

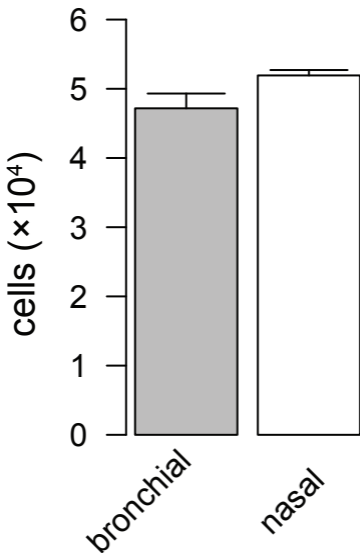

Supplement: S4 Fig — Determined absolute number of cells by DAPI staining within ALI cultures of bronchial and nasal human airway epithelium (see Materials & Methods). The mean ±1.96×.SE over three independent cultures for each condition are shown (S1 Data). (PDF) [file pcbi.1011356.s004.pdf]
