# Supplementary material for: Influence of cell type specific infectivity and tissue composition on SARS-CoV-2 infection dynamics within human airway epithelium
Source: PLoS Comput Biol. 2023 Aug 11;19(8):e1011356. doi: 10.1371/journal.pcbi.1011356 (PMC10446191; doi:10.1371/journal.pcbi.1011356)
Supplement: S1 Table — General parameters for initializing the cellular Potts model with specificities for the different cell types. The xml-files for the standard model for cell differentiation and turnover with the infection dynamics within bronchial tissue, including all baseline parameterizations and implemented in Morpheus 2.2.6, is provided at https://github.com/GrawLab/SARS-ALIculture. (PDF) [file pcbi.1011356.s005.pdf]

S1 Table: Parameters for the cellular Potts model (CPM) of the ALI-culture system. General parameters for initializing the cellular Potts model with specificities for the different cell types. The xml-file for the standard model for cell differentiation and turnover with the infection dynamics within bronchial tissue including all baseline parameterizations and implemented in Morpheus 2.2.6 is provided at https://github.com/GrawLab/SARS-ALIculture.

**Parameter**	**Unit**	**Cell Type**		
		**Basal**	**Secretory**	**Ciliated**
Target volume/area, A_T	μm²	60	60	60
Volume strength, V_S	a.u.	10	100	100
Surface constraint (asphericity,Ψ)	a.u.	0.9	0.9	0.9
Cell-to-cell transmission, relative infection parameter β_{cc}		1	1.536	4.33
Cell-to-cell transmission, relative infecting parameter ρ_{cc}		1	0.5786	0.4859
**Additional parameters for the CPM**				
Viral diffusion rate, D_V	$\mu m^2/h^{-1}$	20		
Simulation timestep size	h	0.25 (infection mdl) / 1.2 (regeneration mdl)		
Effectiveness of cell-free transmission, w_{cf}		N_0/15		
Effectiveness of cell-to-cell transmission, w_{cc}		1/20		
Weighting of initial infection dose, w_V		724.85		
Relative contribution of cell-to-cell transmission, f_{cc}		0.9 [1]		
Fraction of apical layer at which maximal immune protection reached, g_{max}		0.4		
Radius of cell culture	μm	1204		
Total cell number, N_0	cells	47177 (own data, S4 Figure)		

Reference:

[1] Zeng C, Evans JP, King T, Zheng YM, Oltz EM, Whelan SPJ, et al. SARS-CoV-2 spreads through cell-to-cell transmission. Proc Natl Acad Sci U S A. 2022;119(1). doi: 10.1073/pnas.2111400119. PubMed PMID: 34937699; PubMed Central PMCID: PMCPMC8740724
