# Supplementary material for: Influence of cell type specific infectivity and tissue composition on SARS-CoV-2 infection dynamics within human airway epithelium
Source: PLoS Comput Biol. 2023 Aug 11;19(8):e1011356. doi: 10.1371/journal.pcbi.1011356 (PMC10446191; doi:10.1371/journal.pcbi.1011356)
Supplement: S2 Table — Comparison of the different biological processes/aspects considered within the original and extended ODE approach, as well as the spatially resolved CPM. (PDF) [file pcbi.1011356.s006.pdf]

S2 Table: Comparison of ODE and CPM approach. Comparison of the different biological processes/aspects considered within the standard and extended (=final) ODE approach, as well as the spatially resolved CPM.

Aspects considered within the model	ODE		CPM
	standard	extended	
Refractory cell populations	-	x	-
Cell-to-cell transmission	-	-	x
Immune restriction of transmission	-	-	x
