# Supplementary material for: Influence of cell type specific infectivity and tissue composition on SARS-CoV-2 infection dynamics within human airway epithelium
Source: PLoS Comput Biol. 2023 Aug 11;19(8):e1011356. doi: 10.1371/journal.pcbi.1011356 (PMC10446191; doi:10.1371/journal.pcbi.1011356)
Supplement: S3 Table — Composition of tissues at steady-state using the best estimates for the differentiation dynamics inferred from the experimental data [21] (see also Fig 4A/4B). Values are in % of total cell number. (PDF) [file pcbi.1011356.s007.pdf]

S3 Table: Tissue composition at steady-state for different ALI-culture systems. Composition of tissues at steady-state either treated or untreated by cigarette smoking extract (CSE) using the best estimates for the differentiation dynamics inferred from the experimental data [1] (see also **Figure 1** and **Figure 4A/B)**. Values are in % of total cell number.

**Condition**	**Cell Type**		
	**Basal**	**Secretory**	**Ciliated**
bronchial, untreated	39.7	13.4	46.9
bronchial, 2.5% CSE-treatment	51.4	17.5	31.1
bronchial, 5% CSE-treatment	45.4	17.3	37.3
nasal, untreated	17.8	8.7	73.5

References

[1] Schamberger AC, Staab-Weijnitz CA, Mise-Racek N, Eickelberg O. Cigarette smoke alters primary human bronchial epithelial cell differentiation at the air-liquid interface. Sci Rep. 2015;5:8163. Epub 20150202. doi: 10.1038/srep08163. PubMed PMID: 25641363; PubMed Central PMCID: PMCPMC4313097.
